# The Psychological Impact of COVID-19 on Healthcare Providers in Obstetrics: A Cross-Sectional Survey Study

**DOI:** 10.3389/fpsyg.2021.632999

**Published:** 2021-04-09

**Authors:** Lidia Del Piccolo, Valeria Donisi, Ricciarda Raffaelli, Simone Garzon, Cinzia Perlini, Michela Rimondini, Stefano Uccella, Antonella Cromi, Fabio Ghezzi, Maddalena Ginami, Enrico Sartori, Francesca Ciccarone, Giovanni Scambia, Massimo Franchi

**Affiliations:** ^1^Department of Neuroscience, Biomedicine and Movement Sciences, University of Verona, Verona, Italy; ^2^Department of Obstetrics and Gynecology, AOUI Verona, University of Verona, Verona, Italy; ^3^Department of Obstetrics and Gynecology, “Filippo Del Ponte” Hospital, University of Insubria, Varese, Italy; ^4^Department of Obstetrics and Gynecology, University of Brescia, Brescia, Italy; ^5^Division of Gynecologic Oncology, Department of Obstetrics and Gynecology, Catholic University of the Sacred Heart, Rome, Italy

**Keywords:** health care providers, COVID-19, obstetrics, psychological distress, GHQ-12, coping strategies, stress

## Abstract

**Objective:** To assess the psychological distress of healthcare providers (HCPs) working in the field of obstetrics during the coronavirus disease 2019 (COVID-19) pandemic and to identify factors associated with psychological distress at the individual, interpersonal, and organizational level.

**Design:** Cross-sectional survey study.

**Setting:** Four University hospitals in Italy.

**Participants:** HCPs working in obstetrics, including gynecologists, residents in gynecology and obstetrics, and midwives.

**Methods:** The 104-item survey Impatto PSIcologico COVID-19 in Ostetricia (IPSICO) was created by a multidisciplinary expert panel and administered to HCPs in obstetrics in May 2020 *via* a web-based platform.

**Main Outcome Measures:** Psychological distress assessed by the General Health Questionnaire-12 (GHQ-12) included in the IPSICO survey.

**Results:** The response rate to the IPSICO survey was 88.2% (503/570), and that for GHQ-12 was 84.4% (481/570). Just over half (51.1%; 246/481) of the GHQ-12 respondents reported a clinically significant level of psychological distress (GHQ-12 ≥3). Psychological distress was associated with either individual (i.e., female gender, stressful experience related to COVID-19, exhaustion, and the use of dysfunctional coping strategies), interpersonal (i.e., lower family support, limitations in interactions with colleagues), and organizational (i.e., reduced perception of protection by personal protective equipment, perceived delays on updates and gaps in information on the pandemic) factors in dealing with the pandemic.

**Conclusions:** Results confirm the need for monitoring and assessing the psychological distress for HCPs in obstetrics. Interventions at the individual, interpersonal, and organizational level may relieve the psychological distress during the COVID-19 pandemic and foster resilience skills in facing emotional distress.

## Introduction

Since the worldwide outbreak of severe acute respiratory syndrome coronavirus 2 (SARS-CoV-2) in March 2020 (WHO, 2020), healthcare systems and healthcare providers (HCPs) have been placed under extreme pressure and challenges. Different authors outlined the psychological impact of this condition, recommending tailored psychosocial interventions to preserve the well-being of HCPs and the quality of healthcare provided to the patients (Galli et al., 2020; Greenberg et al., 2020; Lai et al., 2020; Nie et al., 2020; Preti et al., 2020; Shaukat et al., 2020; Shreffler et al., 2020; Yao et al., 2020).

North Italian regions were the first in Europe to face the coronavirus disease 2019 (COVID-19) pandemic and the associated pressure on the healthcare system and HCPs (Alfieri et al., 2020; Armocida et al., 2020; Oliva et al., 2020). High levels of burnout, psychological distress, and psychosomatic symptoms were observed in physicians, nurses, and other professionals at the peak of the pandemic (Barello et al., 2020a,b; Giusti et al., 2020; Marton et al., 2020). Although HCPs working with COVID-19 patients reported a higher level of stress, depressive and anxiety symptoms, burnout, and post-traumatic stress disorders than other HCPs (Babore et al., 2020; Di Tella et al., 2020; Trumello et al., 2020), the emergency might have amplified preexisting vulnerability factors for psychological distress, regardless of direct or indirect management of COVID-19 patients. Therefore, baseline risk may help identify those HCPs who are more susceptible to adverse psychological impact of the COVID-19 pandemic. In this regard, HCPs who work in obstetrics are among those with a noticeable baseline risk for burnout and distress (Becker et al., 2006; Govardhan et al., 2012; Wahlberg et al., 2017; Bourne et al., 2019; Slade et al., 2020).

To the best of our knowledge, the data on COVID-19–related psychosocial distress in HCPs in obstetrics are limited, with the exception of a UK-wide study, which identified a high prevalence of depression and anxiety among obstetricians and gynecologists (Shah et al., 2020). HCPs working in obstetrics and gynecology experienced common and unique challenges during the COVID-19 pandemic. Similar to other HCPs, HCPs in obstetrics also had to adjust to the implementation of infection control measures, dedicated “emergency protocols,” personal risk of exposure to infection, as well as concerns about the provision and use of personal protective equipment (PPE) (Alfieri et al., 2020; Armocida et al., 2020; Oliva et al., 2020). Moreover, HCPs in obstetrics faced specific challenges: the limited rescheduling of obstetrics care, the uncertainties about the potential of vertical transmission of SARS-CoV-2, the management of SARS-CoV-2–positive women during labor, the care of psychologically vulnerable patients without the involvement of the partner, and an increased rate of intrauterine fetal death due to reduced use of emergency service (Boelig et al., 2020; Dell'Utri et al., 2020; Franchi et al., 2020; Green et al., 2020; Qiao, 2020; Vafaei et al., 2020; Yalçin Bahat et al., 2020).

Based on this background, we investigated the psychological distress of HCPs working in obstetrics during the current pandemic in different Italian hospitals. This study aimed to identify HCPs with psychological distress and explore potentially associated factors at the individual, interpersonal, and organizational levels. The “socioecological” model proposed by Winkel et al. (2019) explaining how resilience grows in obstetrician-gynecologists was adopted to build up the Impatto PSIcologico COVID-19 in Ostetricia (IPSICO) survey. This model is based on grounded theory and showed that resilience emerges as “a capacity to connect authentically with the work that is influenced by personal and professional surroundings” and underlines the importance of “both individual and collective actions in promoting an environment in which physicians thrive.” Therefore, in our study, we decided to analyze how individual response to adversity (i.e., level of perceived distress) was related either to personal factors (age, gender, psychological well-being before COVID-19 pandemic, perceived risk of infection, coping strategies, professional role, the experience of quarantine or self-isolation, and stressful events related and not related to COVID 19), quality of connections to others inside and outside professional activity (type and quality of support received by family, friends or others, and by colleagues), or to contextual and organizational factors (measures contributing to a greater sense of security, aspects related to the greatest stress, availability of organizational, and clinical protocols to deal with the pandemic). By using this approach, we aim to establish which are the most relevant intervening aspects contributing to the emotional burden of HCPs during the current pandemic and to define the type of intervention that is more appropriate at each level (individual, interpersonal, and organizational). A better understanding of the level at which influencing factors affect the professional well-being of HCPs in obstetrics is highly relevant to guide more appropriate interventions to manage distress and its negative consequences.

## Materials and Methods

### Study Population and Study Design

Target respondents were all HCPs (gynecologists, residents in gynecology and obstetrics, and midwives) working at four Italian University hospitals (the University of Verona, the Catholic University of the Sacred Heart of Rome, the University of Insubria, and the University of Brescia) accruing to a total of 570 HCPs in obstetrics. HCPs were invited by e-mail to complete the IPSICO survey between May 15, 2020, and May 31, 2020. The electronic invitation included the study presentation and the link to the survey located at a web-based platform. Each center provided the complete list of institutional e-mail addresses of target respondents. The survey was administered in the Italian language. Participation was voluntary and anonymous, and no remuneration was offered to respondents. HCPs were reminded up to 3 times by e-mail whether they were willing or not to take part in the survey.

The study was approved by the human research ethics committee of the University of Verona (2020-UNVRCLE-0143469). All participants gave informed consent for study participation and anonymized data collection and analysis for research purposes prior to accessing and completing the survey. There was no funding for the design and conduct of the study.

### The IPSICO Survey

The IPSICO survey was designed and validated by a panel of trainees, specialty tutors, medical educationalists in obstetrics and gynecology, and clinical psychologists of the University of Verona. The survey resulted in a 104-item battery investigating the sociodemographic and professional characteristics of HCPs in obstetrics, the risk appraisal along with perceived social support and coping strategies, the perceived organizational support and changes in the work organization and climate, the emotional impact of COVID-19, and the impact of COVID-19 on the professional life, along with a measure of psychological distress. The survey was composed of validated psychological questionnaires and items tailored to obstetrics practice and COVID-19. Psychological questionnaires were already validated in the Italian language, such as the short version of the Coping Orientation to Problems Experienced (Brief-COPE) (Carver et al., 1989; Carver, 1997; Coolidge et al., 2000) questionnaire, and the General Health Questionnaire-12 (GHQ-12) (Piccinelli et al., 1993; Politi et al., 1994; Goldberg et al., 1997). Newly developed items were limited to exploring sociodemographic, obstetrics, and COVID-19–related factors.

### Variables

The primary outcome was the presence or absence of clinically significant psychological distress in HCPs. The psychological distress level of HCPs was assessed by the validated Italian version of the GHQ-12 (Piccinelli et al., 1993; Politi et al., 1994; Goldberg et al., 1997), a widely used screening instrument for psychological distress. The GHQ-12 was analyzed based on the method proposed by Piccinelli et al. (1993) (all the 12 items at a 4-level scale of the GHQ-12 survey were scored as 0,0,1,1). A Cronbach α of 0.84 indicated a satisfactory internal consistency of the GHQ-12 in our sample (Politi et al., 1994). HCPs reporting a GHQ-12 score ≥3 were considered positive for the presence of clinically significant psychological distress.

A series of individual, interpersonal, and organizational factors have been used to describe the sample and evaluate their associations with the GHQ-12.

Sociodemographic variables included age (continuous variable), gender (i.e., male, female), marital status (i.e., married/cohabitant, separated/widowed, unmarried), family composition (i.e., single, couple, couple with children, two or more adults not familiar), and presence of old parents (i.e., yes, no). Professional variables investigated the professional role (i.e., specialized doctor, trainee doctor, midwife) and the years of work (continuous variable).

Coping strategies were evaluated using the Brief-COPE (Carver et al., 1989; Carver, 1997). The Brief-COPE is composed of 28 items describing different coping strategies self-evaluated by respondents on a 4-point Likert scale ranging from 1 (“not doing it at all”) to 4 (“doing it a lot”). The coping strategies were grouped into emotions-focused (Cronbach α = 0.69), problems-focused (Cronbach α = 0.66), and dysfunctional (Cronbach α = 0.78) coping strategies (Coolidge et al., 2000).

Using categorical variables (i.e., yes, no), the survey has evaluated if participants underwent a quarantine period, experienced a period of self-isolation, or experienced stressful events related and not related to COVID-19. Moreover, categorical variables were used to investigate the adoption of a shift strategy and the availability of organizational and clinical protocols to deal with the emergency problem.

All other variables regarding individual (i.e., psychological well-being before COVID-19; perceived risk of infection and death; level of professional satisfaction before the pandemic; other negative perceptions and feelings related to work—“exhaustion,” “weight of professional role,” “consideration to abandon the professional role,” “working as duty”), interpersonal (i.e., support received by family, friends, trustworthy people, and colleagues; changes in the rules of interaction with colleagues and in the quality of relationship with patients), and organizational factors (i.e., protection by PPE; efficacy of patient triage on admission; utility of the shift strategy; receiving timely and complete information on the pandemic; reduction in the quality of obstetric service and change in perceived obstetric risk; level of involvement as an active part in the reorganization) have been self-evaluated by HCPs on a 10-point Likert scale ranging from 1 (“not at all”) to 10 (“extremely”).

Finally, the respondents were asked to select the aspects related to the greatest stress during COVID-19, the factors associated with a sense of security, the prevailing sensations in the relationship with the patient, and the prevalent feelings toward colleagues. Respondents could give more than one answer selecting the most corresponding ones to their personal experiences.

Further details of the IPSICO survey and details on all the survey variables were reported and described elsewhere (Del Piccolo et al., 2020).

### Statistical Analysis

Descriptive statistics were used to summarize variables collected in the IPSICO questionnaire and the main characteristics of the study population. For the IPSICO questionnaire analysis, no exclusion criteria were used. Descriptive statistics were expressed with mean and standard deviation (SD) for variables with a normal distribution. Non–normally distributed and ordinal variables were described with median and interquartile range (IQR); nominal variables were summarized with numbers and percentages. The GHQ-12 and Brief-COPE's reliability in our sample was estimated on the observed correlations of the items with each other and expressed using the Cronbach α. The Mann–Whitney *U*-test was used to compare two independent groups, and the Kruskal–Wallis test was adopted in the case of three or more categories. Categorical data were analyzed with the χ^2^ test or Fisher exact test as appropriate. All reported *p*-values were two-sided, and significance was considered at *p* < 0.05. In the case of *post-hoc* pairwise comparisons with three or more groups, *p*-values were adjusted using the Bonferroni correction.

After identifying HCPs with psychological distress (i.e., GHQ-12 score ≥3), associated factors at the individual, interpersonal, and organizational levels have been explored using (i) logistic regression models and (ii) comparing HCPs with GHQ-12 score ≥3 with those with GHQ-12 score <3 in terms of perceived aspects related to the greatest stress and of interpersonal and organizational factors.

Logistic regression models were used to investigate the univariate association between clinically significant distress (GHQ-12 score ≥3) and different variables. Variables associated with the dependent variable in univariate analysis were included in a multivariable model, which was developed starting with a backward stepwise selection to eliminate less relevant variables and then using a hierarchical method for the final choice of predictors. The corrected Akaike information criterion was used to select the model (Ratner, 2010; Hosmer et al., 2013; Chowdhury and Turin, 2020). After defining the multivariable logistic regression model with fixed coefficients, multilevel logistic regression analysis was used to explore the multilevel structure of data related to the aggregation of HPCs within University hospitals (Raudenbush and Bryk, 2002). Comparisons between the model with fixed coefficients and multilevel models were made using the corrected Akaike information criterion (Tabachnick et al., 2019). This further analysis was performed because of the fact that our survey data had an inherent multilevel structure: HCPs within University hospitals (van Oyen, 2009). Therefore, the defined multivariable logistic regression model with fixed coefficients would not have completely corrected for between-group (University hospitals) differences, potentially relevant due, for example, to the different incidence of COVID-19 cases in the four geographic areas. The multilevel approach allows exploring effects that vary by hospitals, studying characteristics contributing to this differential effect, and identifying individual–group interaction effects (Cohen et al., 2013). All reported *p*-values were two-sided, and significance was considered at *p* < 0.05.

Data analysis was performed using IBM SPSS Statistics 23.0, Armonk, NY.

## Results

### Sociodemographic, Professional Characteristics, and COVID-19 Impact in the Study Sample

Of 570 invited HCPs, 503 (88.2%) answered the IPSICO survey. Sociodemographic characteristics of the entire study population are summarized in Table 1, overall and stratified by center. The median age of respondents was 34 years (IQR = 29–46 years), and 83.7% of HCPs were female. Midwives represented more than one-third of respondents (38%), followed by trainees (33.6%) and specialized medical doctors (28.4%). Overall, the entire study sample reported a median work experience in the current role of 5 years (IQR = 2–18 years). Regarding family composition, most respondents answered to have a partner, and 38.8% reported to live with children and 7.7% with old parents; 77.7% of HCPs reported to live with someone else. Psychological well-being before the COVID-19 pandemic was self-evaluated as high, with a median value of 8 (IQR = 7–8) on a Likert scale of 1 = “very bad” to 10 = “very good.”

**Table 1:** Sociodemographic and professional characteristics of the study population (*n* = 503).

**Variable**	**Overall**	**Brescia**	**Rome**	**Varese**	**Verona**	***p*-value**
	**503 (100%)**	**185 (36.8%)**	**111 (22.1%)**	**82 (16.3%)**	**125 (24.9%)**	
Age, median (IQR)	34 (29–46)	39 (30–51)^a^	32 (28–38)^b^	35 (29.75–47.25)^a,b^	31 (28–41.5)^b^	<0.001
Gender, *n* (%)						<0.001
Female	421 (83.7)	161 (87.0)^a^	77 (69.4%)^b^	69 (84.1)^a,b^	114 (91.2)^a^	
Marital status, *n* (%)						0.06
Married/cohabitant	278 (55.2)	105 (56.8)	55 (49.5)	53 (64.6)	65 (52.0)	
Separated/widowed	20 (4.0)	12 (6.5)	3 (2.7)	3 (3.7)	2 (1.6)	
Unmarried	205 (40.8)	68 (36.8)	53 (47.7)	26 (31.7)	58 (46.4)	
Family composition, *n* (%)						<0.001
Single	112 (22.3)	33 (17.8)^a^	32 (28.8)^a^	17 (20.7)^a^	30 (24.0)^a^	
Couple	124 (24.7)	36 (19.5)^a^	39 (35.1)^b^	21 (25.6)^a,b^	28 (22.4)^a,b^	
Couple with children	195 (38.8)	87 (47.0)^a^	24 (21.6)^b^	40 (48.8)^a^	44 (35.2)^a,b^	
Two or more adults not familiar	72 (14.2)	29 (15.7)^a,b^	16 (14.4)^a,b^	4 (4.9)^b^	23 (18.4)^a^	
Presence of old parents, *n* (%)	38 (7.6)	21 (11.4)	4 (3.6)	5 (6.1)	8 (6.4)	0.079
Professional role, n (%)						<0.001
Specialized doctor	143 (28.4)	42 (22.7)^a^	54 (48.6)^b^	35 (42.7)^b^	12 (9.6)^c^	
Trainee doctor	169 (33.6)	35 (18.9)^a^	55 (49.5)^b^	24 (29.3)^a,c^	55 (44.0)^b,c^	
Midwife	191 (38.0)	108 (58.4)^a^	2 (1.8)^b^	23 (28.0) ^c^	58 (46.4)^a^	
Years of work experience in the current role, median (IQR)	5 (2–18)	14 (4–22.5)^a^	4 (2–5)^b^	9.5 (2–16.75)^a,c^	5 (2–15.5)^b,c^	<0.001
Psychological well-being before COVID-19, median (IQR)	8 (7–8)	8 (7–8)	8 (7–8)	7 (7–8)	8 (7–8.25)	0.246
Underwent a quarantine period, *n* (%)	51 (10.1)	27 (14.6)	8 (7.2)	5 (6.1)	11 (8.8)	0.079
Experienced a period of self-isolation, *n* (%)	161 (32.2)	84 (45.4)^a^	24 (21.6)^b^	23 (28.0)^b^	31 (24.8)^b^	<0.001
Experience of stressful events related to COVID-19, *n* (%)	261 (51.9)	114 (61.6)^a^	40 (36.0)^b^	35 (42.7)^b,c^	72 (57.6)^a,c^	<0.001
Experience of stressful events *not* related to COVID-19, *n* (%)	118 (23.5)	49 (26.5)	18 (16.2)	18 (22.0)	33 (26.4)	0.180
Perceived risk of being infected, median (IQR)	6 (5–8)	7 (5–8)	6 (4–8)	6 (5–7)	6 (4–8)	0.055
Perceived risk of death in case of infection, median (IQR)	3 (2–4)	3 (2–5)	3 (2–4)	3 (2–5)	3 (2–5)	0.483

*COVID-19, coronavirus disease 2019; IQR, interquartile range. Each subscript letter denotes a subset of University hospital categories whose parameters do not differ significantly from each other at the 0.05 level*.

Concerning the impact of COVID-19, although only 10.1% of HCPs experienced a quarantine period, almost one-third of them (32.2%) decided to undergo a period of self-isolation, and 51.9% of respondents experienced a stressful event related to COVID-19. The perceived risk of infection was reported higher than 5, on a scale of 1–10, by half of the respondents (IQR = 5–8); conversely, the perceived risk of death in the case of infection was lower, with a median of 3 (IQR = 2–4).

### Prevalence of Psychological Distress

**Figure 1:**
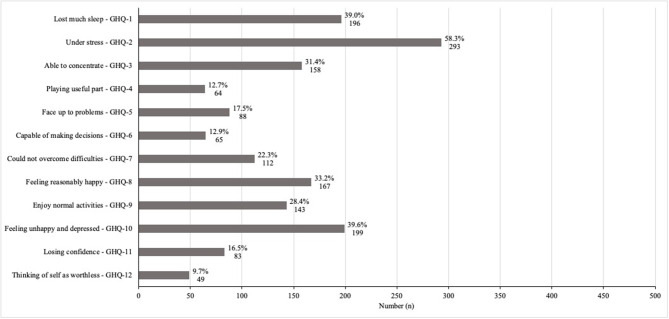
General health and professional well-being assessed with the 12-item General Health Questionnaire (GHQ-12). The proportion of patients with a positive score for a worsening in each item composing the GHQ-12 (*n* = 481).

Four hundred eighty-one of 570 HCPs completed all items of the GHQ-12, resulting in a response rate of 84.4%. The impact of COVID-19 on psychological distress assessed with the GHQ-12 is summarized in Figure 1. GHQ-12 ≥3 was observed in 51.1% of respondents (246/481HCPs).

### Factors Associated With Psychological Distress at the Individual, Interpersonal, and Organizational Levels

#### Factors Associated With Psychological Distress in Univariate and Multivariable Logistic Regression Analyses

Table 2 shows the results of the univariate logistic regression analysis investigating the association between psychological distress (GHQ-12 ≥3) and individual, interpersonal, and organizational factors derived from the IPSICO questionnaire. Variables univariately associated with a GHQ-12 score equal to or higher than 3 were included in the multivariable logistic regression analysis together with age. Individual factors independently associated with a GHQ-12 score ≥3 were gender, the experience of stressful events related to the ongoing pandemic, dysfunctional coping score (Table 3 reports the details regarding the Brief-COPE items composing the dysfunctional coping score) (Carver et al., 1989; Carver, 1997; Coolidge et al., 2000), and perceived exhaustion from work. Interpersonal aspects were the perceived support received from the family and the limited interaction with colleagues. Organizational factors were the perceived protection by PPE and the possibility to receive timely and complete information on the pandemic (Table 4). Multilevel logistic regression models, fitted allowing parameters to vary between the University hospitals (random effect), were compared with the multivariable logistic regression model with fixed coefficients reported in Table 4. The comparison, made with the corrected Akaike information criterion, did not show a statistically significant improvement of the model fit using any multilevel logistic regression model; therefore, we maintained the multivariable logistic regression model with fixed coefficients as it was more parsimonious.

**Table 2:** Univariate logistic analysis of factors evaluated for an association with GHQ-12 ≥3 (*n* = 481).

**Variable**	**Level**	**Univariate**	***p*-value**
		**odds ratio (95% CI)**	
Age	Per 1 year	0.993 (0.977–1.010)	0.414
Years of work experience	Per 1 year	0.991 (0.974–1.008)	0.289
Gender (reference: male)	Female	2.137 (1.285–3.554)	0.003
Marital status (reference: married/cohabitant)	Unmarried	1.114 (0.770–1.611)	0.568
	Separated/widowed	0.716 (0.279–1.838)	0.488
Family composition (reference: single)	Couple	0.564 (0.332–0.959)	0.034
	Couple with children	0.689 (0.425–1.117)	0.131
	Two or more adults not familiar	0.485 (0.263–0.894)	0.020
University hospital (reference: Verona)	Brescia	0.6 (0.374–0.963)	0.034
	Rome	0.467 (0.274–0.797)	0.005
	Varese	0.450 (0.250–0.810)	0.008
Presence of old parents	No	0.770 (0.385–1.543)	0.462
Professional role (reference: midwifes)	Specialized doctor	0.983 (0.630–1.536)	0.941
	Trainee doctors	0.926 (0.608–1.412)	0.722
Underwent a quarantine period	No	1.154 (0.635–2.096)	0.638
Experienced a period of self-isolation	No	0.590 (0.400–0.869)	0.008
Experience of stressful events related to COVID-19	No	0.328 (0.226–0.475)	<0.001
Experience of stressful events not related to COVID-19	No	0.547 (0.355–0.844)	0.006
Psychological well-being before COVID-19	Per 1 point of score	0.840 (0.741–0.952)	0.006
Perceived risk of being infected	Per 1 point of score	1.108 (1.015–1.209)	0.022
Perceived risk of death in case of infection	Per 1 point of score	1.059 (0.957–1.171)	0.269
Support received from my family	Per 1 point of score	0.922 (0.866–0.982)	0.011
Support received from friends and trustworthy people	Per 1 point of score	0.905 (0.847–0.966)	0.003
Emotions-focused coping	Per 1 point of score	1.020 (0.979–1.062)	0.342
Problems-focused coping	Per 1 point of score	1.029 (0.976–1.084)	0.292
Dysfunctional coping	Per 1 point of score	1.120 (1.070–1.173)	<0.001
Perceived protection from PPE	Per 1 point of score	0.884 (0.818–0.955)	0.002
Perceived efficacy of triage for COVID-19 at patient admission	Per 1 point of score	0.887 (0.818–0.962)	0.004
Adoption of a shift strategy to ensure adequate rest and staff always available	No	1.161 (0.798–1.690)	0.435
Utility of the adopted shift strategy	Per 1 point of score	0.983 (0.935–1.034)	0.517
Availability of organizational and clinical protocols to deal with the emergency problem	No	1.548 (0.832–2.882)	0.168
To what extent you received timely and complete information on the pandemic to be able to deal with it adequately	Per 1 point of score	0.820 (0.752–0.895)	<0.001
How much the rules of interaction with colleagues influenced the quality of work	Per 1 point of score	1.153 (1.066–1.247)	<0.001
Perceived reduction in the quality of obstetric service	Per 1 point of score	1.182 (1.097–1.274)	<0.001
Changes in perceived obstetric risk with increased risk of contagion	Per 1 point of score	1.086 (1.010–1.168)	0.026
To what extent the quality of the relationship with the patients has changed	Per 1 point of score	1.171 (1.086–1.263)	<0.001
Level of satisfaction of the profession before the pandemic	Per 1 point of score	0.882 (0.796–0.977)	0.016
I have faced work in this period because it is my duty	Per 1 point of score	1.017 (0.950–1.090)	0.622
Perceived level of involvement as an active part in the reorganization of the activities to deal with the emergency	Per 1 point of score	0.929 (0.867–0.995)	0.034
The entity of perceived support by colleagues who play the same role during the pandemic	Per 1 point of score	0.969 (0.891–1.053)	0.453
The entity of perceived support by the team during the pandemic	Per 1 point of score	0.892 (0.819–0.971)	0.009
A feeling of exhaustion from my job during this pandemic	Per 1 point of score	1.491 (1.363–1.631)	<0.001
The weight of the professional role during this pandemic	Per 1 point of score	1.295 (1.197–1.402)	<0.001
How much was considered to abandon the professional role during this pandemic	Per 1 point of score	1.236 (1.117–1.368)	<0.001

**Table 3:** Details regarding the Brief-COPE items composing the dysfunctional coping factor.

**Brief-COPE items composing the dysfunctional coping factor, *n* (%)**	**1**	**2**	**3**	**4**
I've been turning to work or other activities to take my mind off things.	58 (11.5)	122 (24.3)	220 (43.7)	86 (17.1)
I've been saying to myself, “this isn't real”	291 (57.9)	113 (22.5)	58 (11.5)	24 (4.8)
I've been using alcohol or other drugs to make myself feel better	425 (84.5)	41 (8.4)	14 (2.8)	6 (1.2)
I've been giving up trying to deal with it	332 (66.0)	111 (22.1)	39 (7.8)	4 (0.8)
I've been refusing to believe that it has happened	391 (77.7)	64 (12.7)	21 (4.2)	10 (2.0)
I've been saying things to let my unpleasant feelings escape	207 (41.2)	149 (29.6)	94 (18.7)	36 (7.2)
I've been using alcohol or other drugs to help me get through it	442 (87.9)	27 (5.4)	12 (2.5)	5 (1.0)
I've been criticizing myself	47 (9.3)	125 (24.9)	210 (43.2)	104 (20.7)
I've been giving up the attempt to cope	352 (70.0)	101 (20.1)	32 (6.4)	1 (0.2)
I've been doing something to think about it less, such as going to movies, watching TV, reading, daydreaming, sleeping, or shopping	113 (22.5)	144 (28.6)	151 (31.1)	78 (16.0)
I've been expressing my negative feelings	63 (12.5)	189 (37.6)	182 (36.2)	52 (10.3)
I've been blaming myself for things that happened	417 (82.9)	52 (10.3)	14 (2.8)	3 (0.6)
Dysfunctional coping (mean, SD)	21.38 (4.3)			

*SD, standard deviation. All healthcare provides answered the Brief-COPE; each item provides a score from 1 to 4, which is summed to obtain the dysfunctional coping score*.

**Table 4:** Multivariable logistic regression model of factors evaluated for an association with GHQ-12 ≥3 (*n* = 481).

**Variable**	**Level**	**Univariate odds ratio (95% CI)**	***p* value**
Gender	Male	1.0 reference	
	Female	2.739 (1.482–5.060)	0.001
Experience of stressful events related to COVID-19	Yes	1.0 reference	
	No	0.534 (0.345–0.825)	0.005
A feeling of exhaustion from my job during this pandemic	Per 1 point of score	1.412 (1.279–1.560)	<0.001
Dysfunctional coping	Per 1 point of score	1.070 (1.015–1.127)	0.012
Support received from my family	Per 1 point of score	0.914 (0.847–0.985)	0.018
How much the limitations in interaction with colleagues influenced the quality of work	Per 1 point of score	1.153 (1.047–1.269)	0.004
Perceived protection from PPE	Per 1 point of score	0.883 (0.798–0.977)	0.016
To what extent you received timely and complete information on the pandemic to be able to deal with it adequately	Per 1 point of score	0.850 (0.759–0.952)	0.005
Constant		0.1	0.004

*R^2^ = 0.279 (Cox and Snell), 0.372 (Nagelkerke)*.

#### Association Between Psychological Distress and Perception of HCPs of Aspects Related to the Greatest Stress

The perception of respondents regarding the aspects associated with the greatest stress is shown in Table 5. The fear of infecting the family and the continuous updating of recommendations and measures to be implemented were the most perceived distressing factors. These two aspects related to distress were reported by 56.8% of respondents. However, they were not associated with the GHQ-12 score. The constant and correct use of PPE was the third most frequent aspect related to the greatest stress. It was reported with higher frequency by HCPs in the group having GHQ-12 <3. Conversely, a significantly higher proportion of HCPs in the group having GHQ-12 ≥3 reported difficulties in reconciling private and family life with work, although this aspect was indicated by only 11.6% of HCPs.

**Table 5:** Association between psychological distress and perception of HCPs of aspects related to the greatest stress (*n* = 481).

	**GHQ-12**		
	**<3**	**≥3**	**Total**
	**235 (48.9)**	**246 (51.1)**	**481 (100)**
	**100%**	**100%**	**100%**
Aspects related to the greatest stress in the last period^†^			
Inability to limit routine outpatient activities, *n* (%)	7^a^ (41.20) 3.0%	10^a^ (58.80) 4.1%	17 3.5%
Other, *n* (%)	7^a^ (50) 3.0%	7^a^ (50) 2.8%	14 2.9%
**Reconciling private/family life with work**, ***n*** **(%)**	**17**^**a**^ **(30.40)** **7.3%**	**39**^**b**^ **(69.60)** **15.8%**	**56** **11.6%**
Continuous updating of recommendations and measures to be implemented, *n* (%)	59^a^ (51.8) 25.1%	55^a^ (48.2) 22.4%	114 23.7%
**The constant and correct use of PPE**, ***n*** **(%)**	**52**^**a**^ **(61.9)** **22.1%**	**32**^**b**^ **(38.1)** **13.0%**	**84** **17.5%**
Provide care to an infected patient, *n* (%)	13^a^ (35.1) 5.5%	24^a^ (64.9) 9.8%	37 7.7%
Fear to infect my family, *n* (%)	80^a^ (50.3) 34.0%	79^a^ (49.7) 32.1%	159 33.1%

*χ^2^ Test regarding the entire table = 0.009. In bold significant associations. Each subscript letter denotes a subset of GHQ-12 categories whose column proportions do not differ significantly from each other at the 0.05 level*.

† *Single answer allowed*.

#### Association Between Psychological Distress and Perception of HCPs of Interpersonal and Organizational Factors

Regarding interpersonal and organizational factors at work (Table 6), the group with psychological distress (GHQ-12 ≥3) reported more irritability in the relationship with the patient and guilt about the poor chance of collaboration. The contrary emerged for feelings of group cohesion. For the group having GHQ-12 ≥3, colleagues' support was more frequently reported as a factor that helped to face the emergency at work than in those without the evidence of clinically significant psychological distress (GHQ-12 <3).

**Table 6:** Association between psychological distress and perception of HCPs of interpersonal and organizational factors (*n* = 481).

	**GHQ-12**		
	**<3**	**≥3**	**Total**
	**235 (48.9)**	**246 (51.1)**	**481 (100)**
Measures that gave you a greater sense of security in your relationship with the patient^†^			
Mask worn by the patient, *n* (%)	167^a^ (49.6) 71.1%	170^a^ (50.4) 69.1%	337 (100) 70.1%
Distancing, *n* (%)	66^a^ (55) 28.1%	54^a^ (45) 22.0%	120 (100) 24.9%
Absence of a partner/companion, *n* (%)	56^a^ (46.7) 23.8%	64^a^ (53.3) 26.0%	120 (100) 24.9%
Epidemiological and clinical triage on patient arrival, *n* (%)	98^a^ (49) 41.7%	102^a^ (51) 41.5%	200 (100) 17.5%
Prevailing sensations in the relationship with the patient^†^			
Fear of being infected, *n* (%)	126^a^ (47.5) 53.6%	139^a^ (52.5) 56.5%	265 (100) 55.1%
Fear of infecting someone, *n* (%)	98^a^ (50.8) 41.7%	95^a^ (49.2) 38.6%	193 (100) 40.1%
Difficult communication with the patient due to the absence of a companion, *n* (%)	65^a^ (52.8) 27.7%	58^a^ (47.2) 23.6%	123 (100) 25.6%
**Greater irritability**, ***n*** **(%)**	**18**^**a**^ **(34.6)** **7.7%**	**34**^**b**^ **(65.4)** **13.8%**	**52 (100)** **10.8%**
Reduced tolerance, *n* (%)	30^a^ (42.9) 12.8%	40^a^ (57.1) 16.3%	70 (100) 70.1%
Prevailing feeling that emerged toward colleagues^†^			
Empathy, *n* (%)	70^a^ (48.6) 29.8%	74^a^ (51.4) 30.1%	144 (100) 29.9%
Fear of contagion, *n* (%)	37^a^ (41.6) 15.7%	52^a^ (58.4) 21.1%	89 (100) 18.5%
**Guilt about my poor chance of collaboration**, ***n*** **(%)**	**10**^**a**^ **(29.4)** **4.3%**	**24**^**b**^ **(70.6)** **9.8%**	**34 (100)** **7.1%**
Resentment toward those who avoid exposing themselves to risk, *n* (%)	25^a^ (41) 10.6%	36^a^ (59) 14.6%	61 (100) 12.7%
**Feeling of group cohesion**, ***n*** **(%)**	**96**^**a**^ **(57.5)** **40.9%**	**71**^**b**^ **(42.5)** **28.9%**	**167 (100)** **34.7%**
Feeling of human solidarity in the group, *n* (%)	112^a^ (50.9) 47.7%	108^a^ (49.1) 43.9%	220 (100) 45.7%
Factor that particularly helped to face this emergency at work^†^			
Professional competence, *n* (%)	44^a^ (54.3) 18.7%	37^a^ (45.7) 15%	81 (100) 16.8%
**Support by colleagues**, ***n*** **(%)**	**50**^**a**^ **(41)** **21.3%**	**72**^**b**^ **(59)** **29.3%**	**122 (100)** **25.4%**
Support by family, *n* (%)	36^a^ (43.4) 15.3%	47^a^ (56.6) 19.1%	83 (100) 17.3%
Continuous updating of recommendations and measures to be implemented, *n* (%)	42^a^ (55.3) 17.9%	34^a^ (44.7) 13.8%	76 (100) 15.8%
Constant availability of appropriate contagion containment measures, *n* (%)	48^a^ (57.8) 20.4%	35^a^ (42.2) 14.2%	83 (100) 17.3%
Good relationship with the patient, *n* (%)	25^a^ (52.1) 10.6%	23^a^ (47.9) 9.3%	48 (100) 10%
Passion for my job, *n* (%)	139^a^ (49.8) 59.1%	140^a^ (50.2) 56.9%	279 (100) 58%
High organizational quality of the working context, *n* (%)	16^a^ (47.1) 6.8%	18^a^ (52.9) 7.3%	34 (100) 7.1%
Other, *n* (%)	4^a^ (57.1) 1.7%	3^a^ (42.9) 1.2%	7 (100) 1.5%

*In bold significant associations. Each subscript letter denotes a subset of GHQ-12 categories whose column proportions do not differ significantly from each other at the 0.05 level*.

† *Multiple answers were allowed*.

## Discussion

### Prevalence of Psychological Distress

Half of the HCPs who completed the GHQ-12 reported a clinically significant level of psychological distress (Piccinelli et al., 1993). This result is consistent with previous Italian and international studies exploring the psychological impact of the COVID-19 pandemic on HCPs (Barello et al., 2020a,b; Galli et al., 2020; Giusti et al., 2020; Lai et al., 2020; Nie et al., 2020).

To the best of our knowledge, only one previous study in Europe specifically investigated this topic among HCPs working in obstetrics. Our results confirm the observed high level of psychological distress, although the percentage of HCPs with GAD-2 and PHQ-2 questionnaires scores suggestive of anxiety and depressive disorders was lower in the UK-based study than in our study (i.e., respectively, 25 and 16%) (Shah et al., 2020). However, the comparison with some of the prior studies is limited by using different psychological screening instruments or scoring methods for the GHQ-12. When comparing our results with the studies using the same GHQ-12 scoring system, we observed that the percentage of HCPs with clinically significant psychological distress was similar to that reported in a study conducted in China during the pandemic outbreak (Yao et al., 2020). Moreover, in both studies, “being under stress” and “having lost much sleep” were the GHQ-12 items most negatively affected (Yao et al., 2020). Noteworthy, in our sample, around 40% of HCPs reported being “feeling unhappy and depressed” rather or much more than usual.

Based on our results and previous evidence, psychological screening appears necessary to recognize psychological suffering and prevent negative consequences on HCPs and patient care in obstetrics. Notably, psychological support for these HCPs was already recommended before the current pandemic, considering that gynecologists and midwives are known to be exposed to high levels of posttraumatic stress disorder (Wahlberg et al., 2017; Bourne et al., 2019; Slade et al., 2020).

### Factors Associated With Psychological Distress at the Individual, Interpersonal, and Organizational Levels

In our analysis, the identified model (socioecological) (Winkel et al., 2019), explaining the psychological distress among HCPs in obstetrics, included factors at individual, interpersonal, and organizational levels. Based on the multilevel regression analysis, the relevance of these factors appears similar across the included hospitals. These results reinforce the need to intervene at different levels to reduce the risk of psychological distress in dealing with the COVID-19 pandemic (Winkel et al., 2019; Slade et al., 2020).

#### Role of Individual Factors

During the pandemic, being female was one of the main factors associated with psychological distress in our model, similar to previous studies in obstetrics (Shah et al., 2020) or other HCP categories (Babore et al., 2020; Barello et al., 2020a; Di Tella et al., 2020; Shaukat et al., 2020; Yao et al., 2020). This result is coherent with a higher level of mental and stress disorders observed during the COVID-19 pandemic among females of the general population. This higher vulnerability of females to experience stress and develop posttraumatic symptoms was explained by differences in stress-response systems and a higher involvement as family caregivers (Mazza et al., 2020; García-Fernández et al., 2021). However, the higher prevalence of anxiety and mood disorders in females is recognized in many epidemiological studies (Kessler et al., 2005). Different biological, psychological, social, and gender–role theoretical explanations have been proposed to explain these differences.

Among other individual factors, the professional role and fewer years of experience were not associated with psychological distress in our sample, which is discordant with the previous studies (Barello et al., 2020b; Kisely et al., 2020; Marton et al., 2020; Yao et al., 2020). Conversely, we found that a lower level of self-evaluated psychological well-being before the pandemic was related to psychological distress in univariate analysis. This association was in line with some previous studies in which having a prior history of psychological distress has been considered a vulnerability factor during virus outbreaks (Giusti et al., 2020; Kisely et al., 2020).

Stressful events related to COVID-19 remained a factor associated with psychological distress in the multivariable analysis. Even non-frontline HCPs, such as gynecologists and midwives, experienced highly stressful situations, including quarantine and self-isolation. Notably, consistent with a previous survey in obstetrics (Yalçin Bahat et al., 2020) and HCPs in Italy (Marton et al., 2020), “fear to infect my family” was among the major sources of perceived stress, despite the fact that this fear was not associated with psychological distress.

In terms of coping, higher use of dysfunctional coping strategies was associated with clinically significant psychological distress. This is consistent with previously reported association between avoidant coping strategies and burnout and lower compassion satisfaction in the medical setting (Doolittle, 2020). Moreover, avoidant coping was associated with the perceived stress during the pandemic in an Italian study (Babore et al., 2020). The dysfunctional coping strategies mostly frequently used by our study participants were self-blame but also self-distraction strategies, such as doing something to think about the pandemic less. Notably, in emergency and uncertain conditions such as the COVID-19 pandemic, self-distraction might be considered psychologically protective when used as a short-term strategy, although it might become problematic in the long term.

#### Role of Interpersonal Factors

Higher perceived support by the family was associated with a lower prevalence of psychological distress, in line with previous research among HCPs working in obstetrics (Vafaei et al., 2020; Yalçin Bahat et al., 2020) and other fields (Di Tella et al., 2020; Galli et al., 2020; Kisely et al., 2020; Nie et al., 2020). Our study supports these findings as our participants with clinically significant psychological distress reported twice as those without significant distress “reconciling private and family life with work” as one of the aspects related to the greatest stress.

Moreover, the significance of relationships with colleagues was confirmed as a relevant resilience feature in the obstetrics context (Winkel et al., 2019). Reduced interactions with colleagues were associated with psychological distress. In addition, HCPs presenting with psychological distress showed a higher frequency of “guilt about my poor chance of collaboration” and lower “perception of group cohesion” than the counterparts.

#### Role of Organizational Factors

In line with previous research (Green et al., 2020; Kisely et al., 2020; Nie et al., 2020; Semaan et al., 2020), higher scores in “perceived protection by PPE” and “receiving timely and complete information on the pandemic to deal with it” were associated with lower psychological distress. The relevance of information and PPE on psychological distress in our sample may be intensified because Italy was one of the first countries to face the pandemic. This phase was characterized by continuously changing guidelines and protocols and problematic resource allocation, including PPE, resulting in the exposure of HCPs to safety risk and psychological pressure (Oliva et al., 2020).

Notably, in our sample, HCPs perceived “continuous updating of recommendations and measures to be implemented” as the second aspect related to the greatest stress. This was also a major factor associated with the mental health status in the UK survey on HCPs in obstetrics (Shah et al., 2020).

### Strengths and Limitations

The main strengths of the present study are the high response rate and the inclusion of both gynecologists—whether already specialized or trainees—and midwives. These characteristics allowed building a comprehensive and representative picture of the psychological impact of the COVID-19 pandemic in the obstetrics field. Second, an extensive list of potentially associated factors has been considered in our survey, in line with the conceptualization of psychological distress as a complex interaction between individual, interpersonal, and organizational factors (Winkel et al., 2019). Third, the survey items were created by a panel of experts in the field (i.e., HCPs working in the obstetrics and clinical psychologists supporting hospital HCPs during the pandemic), favoring the feasibility and multidisciplinary nature of the survey. Lastly, the use of validated instruments, such as the GHQ-12, a widely used and validated tool for screening psychiatric morbidity, increases generalizability of our findings and reproducibility of our study by other centers (Goldberg et al., 1997; Werneke et al., 2000).

One of the main limitations to the present study is the cross-sectional design. This study design is not appropriate for determination of causal effect and also limits the ability to explore temporal association and their variations over time. Psychological distress and other HCPs' perceptions and emotions were self-reported simultaneously at a single time point. Therefore, we cannot determine which is a cause and which a consequence of psychological distress. On that basis, further longitudinal studies should be conducted to verify observed results and clarify temporal associations.

Additional limitations related to the cross-sectional design are also present. A non-respondent bias should be considered, although a response rate of 84.4% reduces its impact. A possible referral bias suggests caution in extending our observations to HCPs in obstetrics who do not work in hospitals. In this regard, the generalizability of results may be affected by differences in infection risk and healthcare management of the outbreak across Italy (Armocida et al., 2020; Simione and Gnagnarella, 2020) or worldwide. In terms of geographical context, indeed, HCPs working in regions most affected by the pandemic reported a higher negative psychological impact (Trumello et al., 2020). However, our results suggest that the proposed model does not change across investigated hospitals, given that the multilevel regression analysis did not provide a better model fit in describing the outcome. A recall bias can be present for some items, such as the psychological well-being before the pandemic.

As an additional limitation, the evaluation of “enjoyment of day-to-day activities” of the GHQ-12 may be affected by the Italian lockdown as previously outlined in a UK article (Niedzwiedz et al., 2020). Moreover, our study focused on assessing the psychological distress in the aftermath of the pandemic. Therefore, future studies are required to determine the long-lasting psychological effects and the potential impact on burnout.

## Conclusions

The psychological well-being of HCPs working in obstetrics at four Italian hospitals was poor during the COVID-19 outbreak, given that just over half of the respondents who reported clinically significant psychological distress. This observation stresses the importance of introducing a psychological screening and enhancing individuals and interpersonal and organizational resources to face stressful events, such as a pandemic. At the individual level, psychological interventions should promote acceptance of negative emotions and reduction of avoidance strategies and self-blame and should improve debriefing of stressful experiences. The crucial role of interpersonal factors suggested that group interventions, such as daily experience sharing and peer support, might be effective strategies aimed at normalizing and reducing psychological distress and the perceived difficulties in reconciling private and family life with work. Implementing group initiatives might also enhance the peer recognition of more vulnerable HCPs and reduce stigma. However, at the same time, actions at the organizational level are mandatory to ensure timely and complete access to information and proper material resources, such as PPE. Moreover, at this higher level, a culture of collaboration and support is essential to enhance actions at the individual and interpersonal level, as already suggested for the obstetrics context (Slade et al., 2020). Enhancing these integrated strategies may reduce the psychological impact of COVID-19 and other pandemics and mitigate the potential adverse effects of severe obstetric events, which remain a major source of work-related stress disorders.

## Data Availability Statement

The raw data supporting the conclusions of this article are available from the corresponding author on reasonable request.

## Ethics Statement

The study involving human participants was reviewed and approved by the Human research ethics committee of the University of Verona (CARU, Comitato di Approvazione della Ricerca sull'Uomo) - 2020-UNVRCLE-0143469. The patients/participants provided their informed consent to participate in this study.

## Author Contributions

MF, RR, LD, VD, MR, CP, FG, ES, and GS conceptualized and designed the study. LD, RR, MF, VD, MR, and CP developed the questionnaire. SU, AC, FG, MG, ES, GS, and FC organized and performed the survey. SG, SU, MG, and FC managed the dataset and performed statistical analyses. LD, VD, and SG wrote the manuscript. All the authors conform to the International Committee of Medical Journal Editors (ICMJE) criteria for authorship, contributed to the intellectual content of the study, approved the final version of the article, contributed to the interpretation of the results, and the writing and editing of the manuscript.

## Conflict of Interest

The authors declare that the research was conducted in the absence of any commercial or financial relationships that could be construed as a potential conflict of interest.
